# Reconstruction of Zika Virus Introduction in Brazil

**DOI:** 10.3201/eid2301.161274

**Published:** 2017-01

**Authors:** Kate Zinszer, Kathryn Morrison, John S. Brownstein, Fatima Marinho, Alexandre F. Santos, Elaine O. Nsoesie

**Affiliations:** Boston’s Children’s Hospital, Boston, Massachusetts, USA (K. Zinszer, J.S. Brownstein);; McGill University, Montreal, Quebec, Canada (K. Morrison);; Harvard Medical School, Boston (J.S. Brownstein);; Ministry of Health, Brasilia, Brazil (F. Marinho, A.F. Santos);; University of Washington, Seattle, Washington, USA (E.O. Nsoesie)

**Keywords:** Zika, Zika viruses, viruses, epidemics, introduction, speed, spatial analysis, modeling, Brazil

## Abstract

We estimated the speed of Zika virus introduction in Brazil by using confirmed cases at the municipal level. Our models indicate a southward pattern of introduction starting from the northeastern coast and a pattern of movement toward the western border with an average speed of spread of 42 km/day or 15,367 km/year.

Autochthonous transmission of Zika virus has been confirmed in 67 countries worldwide and in 46 countries or territories in the Americas (*1*,*2*). It is believed that Zika virus was introduced into the Americas through Easter Island in 2014, after an outbreak in French Polynesia (*3*,*4*). Despite the rapid spread of Zika virus across the Americas and global concerns regarding its effects on fetuses, little is known about the pattern of spread. The risk for local transmission in unaffected regions is unknown but potentially serious where competent Zika virus vectors are present (*5*) and also given the additional complexities of sexual transmission and population mobility (*3*,*6*).

Knowledge of the direction and speed of movement of a disease is invaluable for public health response planning, including timing and placement of interventions. We estimated the speed of Zika virus spread in Brazil by using data on confirmed cases of Zika virus disease at the municipal level and applying an approach used in estimating the speed of Ebola spread across parts of West Africa (*7*).

## The Study

Confirmed cases of Zika virus disease were obtained from the Brazil Ministry of Health. Additional reports were also extracted from ProMED mail (*8*) and HealthMap (*9*). We performed the analysis by using 3 dates: 1) date of case registration in the surveillance system of the Brazilian Ministry of Health (model 1); 2) earliest of either date of symptom onset (if available) or registration date (model 2); and 3) earliest of either case registration date, date of symptom onset, or date of case report by other sources (model 3). Surface trend analysis was used to interpolated a continuous estimate of disease spread speed in magnitude and direction (*10*) by using available spatial and temporal information. Time of dispersal was calculated from the start of the epidemic for each model (Technical Appendix).

**Figure 1:**
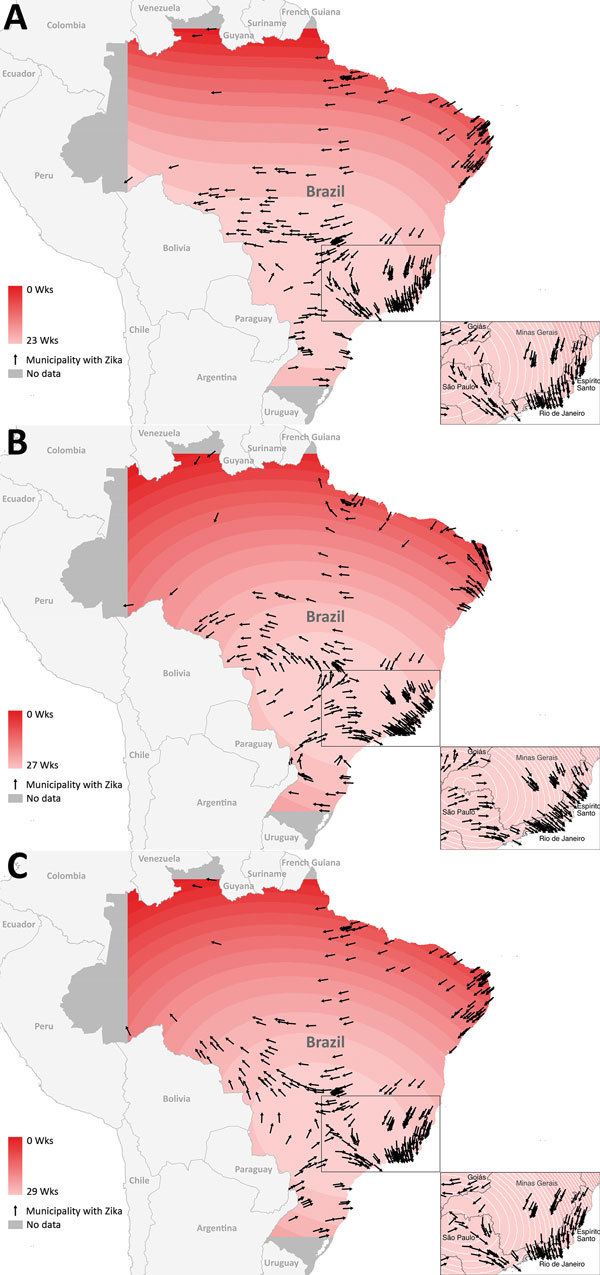
Contour surface trends and directional vectors for reconstructing Zika introduction in Brazil. A) Date of case registration (model 1); B) earliest date between date of symptom onset (if available) and date of registration (model 2); C) earliest date between date of case registration, date of symptom onset, and date of case reporting by other sources (model 3). Each contour line represents a 1-day period, and contour lines farther apart show that the disease spread rapidly through an area, whereas lines close together show slower progression in an area. Arrows indicate direction of Zika spread. Magnitude of speed and direction should be interpreted cautiously near the edges of the study area. Estimates of speed are subject to edge effects, which indicates that estimates are less stable because they are based on fewer data (not as many neighboring values).

Data provided by the Brazilian Ministry of Health on May 31, 2016, indicated that Zika had been confirmed in 316 of 5,564 municipalities in 26 states; 6 additional municipalities were identified from other reporting sources. Contour maps of interpolated temporal trends (Figure 1) indicate a trend of spread into southern and western Brazil, and initial outbreak reports originated from municipalities along the northeastern coast. On the basis of confirmed cases, the earliest location of spread was the northeastern coastal area between the states of Paraíba, Ceará, Bahía, Alagoas, and Rio Grande do Norte. There were also earlier dates of self-reported symptom onset in the northwestern state of Amazonas (January 1, 2015), the west-central state of Matto Grosso (January 4, 2015), and the southeastern coastal state of Rio de Janeiro (January 1, 2015).

Contour maps (Figure 1) indicate slight differences in patterns of dispersion between the models. Model 1 indicates the strongest trend of a southward spread from the northeastern coast toward the populous southeastern coastal states of Rio de Janeiro, Espírito Santo, and São Paulo; the estimated time of dispersal was 22 weeks (Figure 1, panel A). In addition to west to east spread of Zika in southern Brazil, there was a pattern of movement west toward Bolivia.

The dispersal trend for model 2 was more varied but also indicated spread to the southeastern coastal states of Rio de Janeiro, Espírito Santo, and São Paulo (Figure 1, panel B). This model also suggests an initial spread north from the earliest reports in the northeastern region and a spread west toward Bolivia. The model estimates a north to south diffusion of ≈27 weeks. Model 3 suggests a strong southward spread originating from the northeastern coast toward the southeastern coastal states (approximate dispersal time of 29 weeks) and toward the western border and northwestern state of Amazonas (Figure 1, panel C).

**Figure 2:**
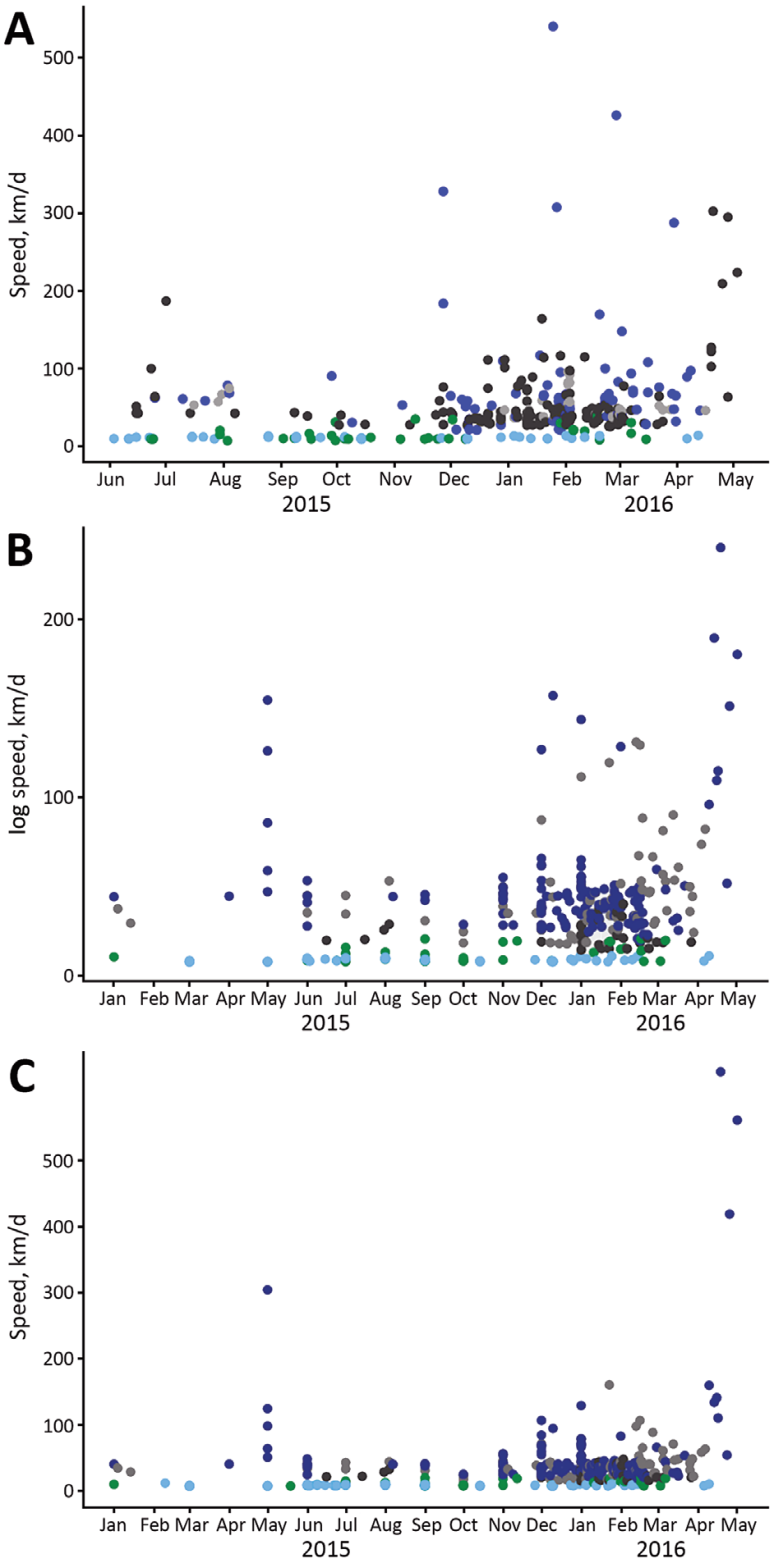
Speed or log speed (km/d) of Zika introduction into municipalities in Brazil. A) June 2015–May 2016; B) January 2015–May 2016; C) January 2015–May 2016. Municipalities are classified by region. Gray circles indicate central–western region, green circles indicate northern region, light blue circles indicate northeastern region, black circles indicate southern region, and dark blue circles indicate southeastern region.

Overall, the average speed of diffusion was 42.1 km/day or 15,367 km/year. The minimum speed across all 3 models was 6.9 km/day, and the maximum speed was 634.1 km/day (Figure 2). Municipalities in northeastern and northern regions had the slowest speeds, and municipalities in the west-central and southeastern regions had the highest speeds. This finding was caused by proximity of cases in time and space. More cases occurred closer in time and over larger areas in southern, southeastern, and west-central regions, which resulted in faster rates of case introduction.

All models were consistent in agreement that Zika dispersal in Brazil followed a general pattern of southward spread toward the populous coastal states (average speed of introduction of 42 km/day), which could be explained by multiple introductory cases into different areas probably caused by movement of viremic persons. We estimate that it took ≈5–6 months for Zika to spread from the northeastern coast to the southeastern coast and western border of Brazil. These findings are supported by the first report of local transmission of Zika virus in Paraguay in late November 2015 (*11*) and in Bolivia in January 2016 (*12*), 7 months after the first registered case in Brazil.

Limitations of this analysis include quality and timeliness of surveillance data that provided the basis for this study. Symptom onset date is subject to error because it is based on self-report, and earlier introductions of Zika in some municipalities might not have been captured by the Ministry of Health surveillance system and supplementary data sources, given the mild and generic nature of Zika symptoms and the high proportion of asymptomatic persons (*3*). The northern region of Brazil had a major dengue outbreak in early 2015, and given symptom similarities between dengue and Zika, it is probable that some suspected dengue cases were in fact early cases of Zika.

Sporadic geographically disparate cases were recorded in various parts of Brazil, which increased the uncertainty associated with speed analysis. These cases, such as those in northwestern Brazil, increased uncertainty in direction and speed estimates, which are also related to edge effects. Edge effects occurred along the boundary of the study area, which in this study were constructed by using fewer data points and are therefore less stable. This effect is shown with directional arrows pointing toward earlier areas of spread versus toward later areas of spread (Figure 1, panels B, C).

## Conclusions

The arrival and rapid spread of Zika virus in the Americas resembles that of chikungunya virus, which was introduced into Saint Martin in the Caribbean in 2013 (*13*,*14*). Increased knowledge of the speed of spread and direction of Zika spread can help in understanding its possible future directions and pace at which it travels, which would be essential for targeted mosquito control interventions, public health messages, and travel advisories. Future work will investigate underlying causes for the southward and westward spread in Brazil by incorporating mobility data and seasonal events, such as movement of persons between northeastern and southeastern regions for vacations, which could have driven the spatial transmission pattern. Furthermore, multicountry analysis is needed to understand continental spatial and temporal patterns of dispersion of Zika virus and co-circulating viruses, such as chikungunya virus.

Technical Appendix Additional information on reconstructing Zika introduction in Brazil.
