# Supplementary material for: Reconstruction of Zika Virus Introduction in Brazil
Source: Emerg Infect Dis. 2017 Jan;23(1):91–4. doi: 10.3201/eid2301.161274 (PMC5176213; doi:10.3201/eid2301.161274)
Supplement: Technical Appendix — Additional information on reconstructing Zika introduction in Brazil. [file 16-1274-Techapp-s1.pdf]

Article DOI: http://dx.doi.org/10.3201/eid2301.161274

Reconstruction of Zika Virus Introduction in Brazil

Technical Appendix

Suspected Zika cases, on the basis of the Pan American Health Organization and the World Health Organization definition (*1*), were confirmed by using reverse transcription PCR (99% of cases) and virus isolation in Vero or C6-36 cell lines. In addition to Ministry of Health data, Zika case data were obtained from HealthMap (*2*) and ProMED mail (*3*). HealthMap is an internet disease surveillance system based on media and outbreak reports from official public health sources (*2*), and ProMED mail is a moderated communication network for outbreak reporting and information exchange (*3*). Six additional municipalities were identified through the use of HealthMap and ProMED mail. From these data, we identified the registration date (date of entry into the surveillance system) of the first official confirmed Zika case in each municipality, the first symptom onset date for all confirmed cases, and any additional confirmed case dates from either HealthMap or ProMED mail.

Centroids of municipalities in Brazil were taken in meters from shapefiles and used to perform surface trend analysis. These data were geocoded by joining them to shapefiles for the municipalities (Universal Transverse Mercator zone 23 South projection) obtained from the DIVA Geographic System (*4*). Surface trend is a spatial interpolation method used to estimate continuous surfaces from point data. Traditionally, it has been used to model geographic elevation, but it also has been used to generate contour lines for representing disease spread across geographic space (*5,6*).

The response variable was time in days from first Zika case for each coordinate, which was January 1, 2015, for the earliest date between symptom onset and registration date (model 2); the earliest date between symptom onset, registration, or other case report (model 3); and June 3, 2015, for the registration date (model 1). The continuous surface of time to infection was estimated by regressing it against the X and Y coordinates. Time was in days and X and Y coordinates were meters. Parameters were estimated by using least squares regression, and if a

simple 2-dimensional plane through the points is insufficient to model the data, high-order polynomials are often used to capture local scale trends (*6*,*7*). We estimated models beginning with only linear terms by $f(t|X,Y) = \beta_0 + \beta_1 X + \beta_2 Y + \varepsilon$, where $E(\varepsilon) = 0$ (Equation 1).

We explored 10 models by incrementally adding polynomials up until the order 10. Every model beyond the linear model reported by the Pan American Health Organization (*1*) included an interaction term between X and Y:

$$f(t|x,y) = \beta_0 + \beta_1 X + \beta_2 Y + \beta_3 X^2 + \beta_4 Y^2 + \ldots + \beta_{19} X^{10} + \beta_{20} Y^{10} + \beta_{21} XY + \varepsilon \text{ (Equation 2).}$$

The best-fit model was selected by using R^2. The model with polynomial terms of order 3 provided the best fit for the registration date, and polynomial order 2 for the earliest date between symptom onset and registration date, and the earliest date between symptom onset, registration, or other case report.

The residuals for each of the models were assessed for spatial autocorrelation, but no significant correlation was observed in models beyond the linear model in (*1*). The rate of change was obtained by taking the partial derivatives with respect to X and Y, for the best-fit linear model, shown below as order 3 polynomial (*3*):

$$f(t|x,y) = \beta_0 + \beta_1 X + \beta_2 Y + \beta_3 X^2 + \beta_4 Y^2 + \beta_5 X^3 + \beta_6 Y^3 + \beta_7 XY \text{ (Equation 3);}$$

$$\partial f(t|x,y)/\partial x = \beta_1 + 2\beta_3 X + 3\beta_5 X^2 + \beta_7 Y \text{ (Equation 4);}$$

$$\partial f(t|x,y)/\partial y = \beta_2 + 2\beta_4 Y + 3\beta_6 Y^2 + \beta_7 X \text{ (Equation 5).}$$

Equations 4 and 5 provide expressions for a slope vector at a given location (X,Y). The vectors can be converted to express the magnitude and direction of rate of change (in days per kilometer) by finding the inner product of the vector, where magnitude $||xy|| = \sqrt{(x^2 + y^2)}$ and the direction $\theta = \tan^{-1}(y/x)$. Note that care must be used when applying the directions of the vectors (such as for vector field mapping); thus, the correct reference axis is used. The rate we were primarily interested in was speed (kilometers per day), which we obtained by inverting the final magnitude of the slope.

References

1. Pan American Health Organization. Case definitions [cited 2016 Jun 30]. http://www.paho.org/hq/index.php?option=com_content&view=article&id=11117&Itemid=41532&lang=en

2. Brownstein JS, Freifeld CC, Reis BY, Mandl KD. Surveillance Sans Frontières: Internet-based emerging infectious disease intelligence and the HealthMap project. PLoS Med. 2008;5:e151. http://dx.doi.org/10.1371/journal.pmed.0050151

3. Madoff LC. ProMED-mail: an early warning system for emerging diseases. Clin Infect Dis. 2004;39:227–32. http://dx.doi.org/10.1086/422003

4. DIVA GIS. Free spatial data [cited 2016 Mar 17]. http://www.diva-gis.org/Data

5. Berrang-Ford L, Berke O, Abdelrahman L, Waltner-Toews D, McDermott J. Spatial analysis of sleeping sickness, southeastern Uganda, 1970–2003. Emerg Infect Dis. 2006;12:813–20. http://dx.doi.org/10.3201/eid1205.051284

6. Pioz M, Guis H, Calavas D, Durand B, Abrial D, Ducrot C. Estimating front-wave velocity of infectious diseases: a simple, efficient method applied to bluetongue. Vet Res. 2011;42:60. http://dx.doi.org/10.1186/1297-9716-42-60

7. Ball FG. Front-wave velocity and fox habitat heterogeneity. In: Bacon PJ, editor. Population dynamics of rabies in wildlife. New York: Academic Press; 1985. p. 255–89.
